# Involvement of Indigenous young people in the design and evaluation of digital mental health interventions: a scoping review protocol

**DOI:** 10.1186/s13643-021-01685-7

**Published:** 2021-05-05

**Authors:** Josie Povey, Buaphrao Raphiphatthana, Michelle Torok, Tricia Nagel, Fiona Shand, Michelle Sweet, Anne Lowell, Patj Patj Janama Robert Mills, Kylie Dingwall

**Affiliations:** 1 Menzies School of Health Research, Charles Darwin University, Ellengowan Drive, Darwin, Northern Territory 0909 Australia; 2 University of New South Wales, Sydney, New South Wales 2052 Australia; 3 Menzies School of Health Research, Adelaide Campus, Adelaide, 0810 Australia; 4 Charles Darwin University, Research Institute for Environment and Livelihoods, Ellengowan Drive, Darwin, Northern Territory 0909 Australia; 5 Menzies School of Health Research, Cnr Simpson and Skinner Street, Alice Springs, Northern Territory 0871 Australia

**Keywords:** Digital mental health, Indigenous, Adolescent, Young adult, Scoping review, Participatory

## Abstract

**Background:**

Indigenous young people worldwide are at greater risk of developing mental health concerns due to ongoing inequity and disadvantage. Digital mental health (dMH) interventions are identified as a potential approach to improving access to mental health treatment for Indigenous youth. Although involvement in the development and evaluation of dMH resources is widely recommended, there is limited evidence to guide engagement of Indigenous young people in these processes. This scoping review aims to examine the methods used to involve Indigenous young people in the development or evaluation of dMH interventions.

**Methods:**

Articles published in English, involving Indigenous young people (aged 10–24 years) in the development or evaluation of dMH interventions, originating from Australia, New Zealand, Canada and the USA will be eligible for inclusion. PubMed, Scopus and EBSCOhost databases (Academic Search Premiere, Computer and Applied Science complete, CINAHL, MEDLINE, APA PsychArticles, Psychology and Behavioural Sciences collection, APA PsychInfo) will be searched to identify eligible articles (from January 1990 onwards). Infomit and Google Scholar (limited to 200 results) will be searched for grey literature. Two reviewers will independently screen citations, abstracts and full-text articles. Study methods, methodologies, dMH intervention details, participant information and engagement, and dissemination methods will be extracted, analysed (utilising content analysis), and qualitatively assessed for alignment with best practice ethical guidelines for undertaking Indigenous health research. A narrative summary of findings will be presented. Reporting will follow the Consolidated Criteria for Strengthening Reporting of Health Research involving Indigenous peoples (CONSIDER) and the Preferred Reporting Items for Systematic Reviews and Meta-Analyses extension for scoping reviews (PRISMA-ScR) guidelines.

**Discussion:**

To date, there are no reviews which analyse engagement of Indigenous young people in the development and evaluation of dMH interventions. This review will appraise alignment of current practice with best practice guidelines to inform future research. It will highlight appropriate methods for the engagement of young people in study processes, providing guidance for health practitioners, policy makers, and researchers working in the field of Indigenous youth and dMH.

**Systematic review registration:**

Open Science Framework (osf.io/2nkc6).

**Supplementary Information:**

The online version contains supplementary material available at 10.1186/s13643-021-01685-7.

## Background

The majority of Indigenous young people worldwide are resilient, proud of their culture, and possess social capital beyond any other recent generation [1–3]. Despite this, they remain at heightened risk of developing mental illness in adolescence compared with their non-Indigenous counterparts [4]. We use the collective term ‘Indigenous’ to describe people who originate from a particular region but acknowledge the rich diversity of cultures and knowledge represented by this term. Despite the need, Indigenous young people worldwide are less likely to access mental health treatment than non-marginalised young people [5, 6]. Barriers to accessing mental health treatment include stigma, fear, shame, intergenerational trauma, distrust of services and being unable to identify signs and symptoms of illness [5, 6]. Furthermore, the location of populations in need are often decentralised, meaning long distances and increased costs and challenges in the delivery of services [5, 7]. Treatment services are often non-existent, underfunded or occur in a localised or prescribed manner within Indigenous communities, limiting their ability to affect meaningful sustained change. Considerations of language, diversity and worldview differences are sometimes overlooked, resulting in programs that are less meaningful or acceptable to the young people they are intended to serve [8, 9]. Despite the need for culturally safe, effective and early intervention treatments, there remain relatively few approaches which are evidence-based. Recent increased availability of technology and connectivity has been identified as an opportunity to increase access to health services within underserved communities and marginalised youth populations [6].

dMH is identified as “mental health, suicide prevention and alcohol and other drugs services delivered via a digital platform” [10]. There remains limited adaptation to cultural diversity in the dMH field, despite the potential of dMH to increase access to treatment for Indigenous populations [11–13]. To date, five systematic or scoping reviews have been conducted in the area of health technologies for Indigenous populations [14–18] but only one has focused on young people and mental health interventions [18]. One other related article contains a literature review and a case study [19]. There is consensus among the authors of these reviews that meaningful engagement of end users in design, development or evaluation is a necessary component of digital health solutions [14, 16–18]. However, none comprehensively examine the strategies undertaken to engage Indigenous young people in the development or evaluation process.

Despite widespread recognition of the importance of end-user involvement in the development and evaluation of dMH interventions [10, 20, 21], there remains a lack of clear reporting on the methods and processes undertaken [22, 23]. A popular methodology outlined in the literature is Participatory Design (PD), which involves end users in the co-creation and evaluation of digital health resources as partners [24]. This process allows an iterative approach whereby digital resource design is continually reviewed and updated throughout all design, development and evaluation phases [21], as recommended in the National Safety and Quality Digital Mental Health Standards [10].

Furthermore, ethical guidelines on the conduct of research with Indigenous communities emphasise the importance of cultural considerations when engaging Indigenous people in research practices [25–29]. Most of these guidelines highlight the importance of (1) accessible, clear and co-constructed benefits to the community or individual, (2) meaningful relationships, consultation and participation of Indigenous people within all stages of research processes, (3) respecting and upholding Indigenous knowledge and practices and (4) Indigenous self-determination and governance [25, 27–30]. Meaningful engagement of Indigenous youth potentially protects young people and communities from being detrimentally affected and disempowered and allows better opportunity for self-determination [25, 31, 32]. Critically reviewing research practices ensures ethical guidelines are upheld and safeguards and informs the best practice into the future [31]. Such reviews have resulted in specific guidelines, such as the Consolidated Criteria for Strengthening Reporting of Health Research involving Indigenous peoples (CONSIDER statement), which aims to improve the quality of reporting of research practices involving Indigenous peoples [33]. Guidelines or recommendations for the involvement of Indigenous young people in dMH development or evaluations do not currently exist.

International research has identified strengths unique to Indigenous young people which help build and maintain resilience [34, 35]. Strong connections with land, culture, lore, family and community assist Indigenous young people to develop strong identities, physical health, mental health and well-being. These connections provide the foundation for healthy development into adulthood [34, 36–38]. Their unique strengths, coupled with an affinity for creative technological innovation and design [14, 39, 40], and willingness to embrace empowerment and self-determination [1] provide a strong foundation for the involvement of Indigenous young people in the development and design of dMH interventions [41]. To ensure an ethical and culturally responsive process, it is essential to understand best practice methods for the engagement of Indigenous young people in the development and evaluation of dMH solutions. Therefore, the objectives of this review are to comprehensively synthesise research reporting on the methods used to involve Indigenous young people (aged 10 to 24) in the development or evaluation of dMH interventions and to examine the degree to which those methods align with the best practice ethical guidelines for undertaking Indigenous health research [26–28, 30].

## Methods

### Study design

Scoping reviews are particularly useful in providing an overview of research on a given topic where evidence is emerging [42] and to review research processes on a given topic [43]. For these reasons, a scoping review was considered the most appropriate methodology. This scoping review will be conducted according to guidelines proposed by Arksey and O’Malley [44] and the subsequent modifications proposed by Levac et al. [42] and Peters et al. [45]. It involves a six-stage process which includes (1) identifying the research question; (2) identifying relevant studies; (3) developing a study selection and data extraction method, which is refined using an iterative process [42]; (4) charting the data; and (5) collating, summarising and reporting results. Additionally, Step 6, consultation, engages two senior Indigenous researchers (PPRJM) throughout scoping review processes, a minimum of three times, ensuring analysis and findings are informed by Indigenous worldviews. Given the iterative nature of a scoping review, changes to the protocol can be expected [45], and any changes are detailed and justified in the final reporting.

### Protocol registration and reporting information

This scoping review protocol is being reported in accordance with the Preferred Reporting Items for Systematic Reviews and Meta-Analyses Protocols (PRISMA-P) statement (see Additional file 1) and has been registered with Open Science Framework (registration identifier: osf.io/2nkc6). The proposed scoping review will be reported in accordance with the reporting guidance provided in the Preferred Reporting Items for Systematic Reviews and Meta-analyses (PRISMA) extension for Scoping Reviews (PRISMA-ScR) [46].

### Eligibility criteria

#### Type of studies

All primary research study designs will be eligible for inclusion, such as experimental studies (e.g. randomised controlled trials, non-randomised controlled trials), observational studies (e.g. cross-sectional studies, cohort studies), quasi-experimental studies and qualitative studies. Systematic reviews, meta-analyses, opinion pieces and narrative reviews will be excluded.

### Participants

Studies involving Indigenous young people, originating from Australia (Aboriginal and Torres Strait Islander), New Zealand (Maori), Canada (Inuit, First Nations people) and the USA (First Nations people) are eligible for inclusion. Indigenous people in these developed first world countries have experiences of colonisation, persistent health inequities and notable rural and remote residence. In addition, Indigenous people’s health worldviews, language and cultural needs differ from mainstream populations. Young people, for the purposes of this review, refer to those who are aged 10−24 years, representative of a broader definition of adolescence, as described by Sawyer et al. [47].

### Intervention

dMH services are identified as “mental health, suicide prevention and alcohol and other drugs services delivered via a digital platform”, which include the “provision of information, digital counselling services, treatment services (including assessment, triage and referral services) and peer-to-peer support services” [10]. Some examples of dMH services for Indigenous people include interventions delivered via smartphone applications [48], therapist-supported digital interventions on tablet devices [49], online CBT programs [50] and gamified CBT interventions [51]. For the purposes of this review, we will include studies reporting on the design, development or evaluation of mental health interventions, which use a digital platform (e.g. smartphone, tablet device, website, computer, wearable devices) to deliver mental health services (e.g. health promotion/psycho-education, prevention/early intervention, crisis intervention/suicide prevention, treatment, recovery and mutual/peer support). Studies describing interventions such as telepsychiatry and videopsychiatry without the significant use of other computerised methods (e.g. websites, online game or SMS support) will not be eligible, as these services are more closely aligned with face-to-face services than dMH services which rely on computerised systems and are designed to be less resource dependent and easily replicated to the wider population [52, 53]. The primary treatment focus of the dMH intervention must be improvement of mental health or well-being outcomes, which include psychological distress, anxiety/stress management, suicidality, substance use and smoking, to be eligible for inclusion. Studies with a primarily physical health focus (e.g. diabetes, HIV management) will be excluded. Electronic health or medical records, decision support tools for clinicians, analytic services, services that primarily provide support and education to health professionals, clinical practice management software, and clinical workflow and communication software are excluded [10]. A full list of inclusion and exclusion criteria is included in Table 1.

**Table 1:** Inclusion and exclusion criteria

Inclusion criteria	
• Minimum 50% of study participants are identified as Indigenous • Minimum 50% of study participants are aged 10–24 years • Studies based in Australia, Canada, New Zealand and the USA • Interventions targeting the mental health of young people (including health promotion/psycho-education, prevention/early intervention, crisis intervention/suicide prevention, treatment, recovery and mutual/peer support) • Young people are involved in dMH design, development and/or evaluation • Interventions delivered using Information Communication Technology (smartphone, iPad, websites, computers and other digital devices) • Primary focus of the study is mental health problems and/or well-being outcomes, including suicidality, substance use, and smoking.	
Exclusion criteria	
• Not related to mental health/well-being (i.e. physical health as outcome) • Study population outside of above culture, age and geographic parameters • Young people are not involved in design or evaluation or are not the intended target audience of the dMH intervention • Non-English language studies (due to limitations in time/resources) • Studies focused on telepsychiatry via videoconferencing or telephone; without a significant engagement with apps, websites, email or other computerised systems • Electronic health or medical records, decision support tools for clinicians, analytic services, services that primarily provide support and education to health professionals, clinical practice management software, and clinical workflow and communication software	

### Outcomes

There are two broad categories of outcomes that are of interest: (1) study methodology and methods and (2) alignment of methods with relevant ethical guidelines. Specifically, methods will be assessed in terms of accessible, clear and co-constructed benefits to the community or individual; meaningful relationships, consultation and participation of Indigenous people within all stages of research processes; respect and recognition of Indigenous knowledge and practices and Indigenous self-determination and governance [27–30].

### Information sources and search strategy

Following recommendations by Joanna Briggs Institute, a three-step search process will be used. First, two independent reviewers (JP, BR) will undertake a limited search of the databases (EBSCOhost and PubMed). Search terms for EBSCOhost database are included in supplementary file 2. Titles, abstracts and keywords of retrieved articles will be reviewed to find additional search terms, before three reviewers (JP, BR, MT) meet to finalise keywords. Search terms will then be updated. The primary source of literature will be a structured search of multiple electronic databases from January 1990 onwards—considering the emergence of the internet in the mid 1990’s provided opportunities for health professionals to explore alternatives to face-to-face care [54]. Databases will include EBSCOhost databases (Academic Search Premiere, Computer and Applied Science complete, CINAHL Plus with Full text, MEDLINE with full text, APA PsychArticles, Psychology and Behavioural sciences collection, APA PsychInfo); PubMed; Scopus. The secondary source of potentially relevant material will be a search of the grey or difficult-to-locate literature, including Informit and Google Scholar (limited to the first 200 results). We will perform hand-searching of the reference lists of included studies, relevant reviews, or other relevant documents. Content experts and authors who are prolific in the field will be contacted. If full text is not available, corresponding authors will be contacted.

### Screening and selection procedure

First, the titles and abstracts of articles returned from initial searches will be screened based on the eligibility criteria outlined above. Two reviewers (JP, BP) will independently review a 10−15% subset of the complete set of search results to establish a good inter-rating agreement (defined as kappa of 0.81 or above). Once good inter-rater reliability is established, one reviewer (JP) will then continue to screen the rest of the articles before proceeding to full-text review. The same procedure will be carried out for the second screening stage using full texts. In the third stage, references of all considered articles will be hand-searched to identify any relevant studies missed in the search strategy. Any disagreements will be resolved by discussion to meet a consensus, if necessary. If consensus is not reached throughout each stage of study selection, a third reviewer (MT) will review the articles in question. A flow chart showing details of studies included and excluded at each stage of the study selection process will be provided [46].

### Data extraction

Data extraction variables, outlined in Table 2, are converted into simple tables prior to data extraction. Data extraction forms will be independently tested by two reviewers (JP, BR) on a random sample of five studies to ensure accuracy, consistency and validity of captured information [42, 55]. One reviewer (JP) will then extract relevant data. If data are missing upon review of full text, corresponding authors will be contacted.

**Table 2:** Data extraction variables

Variable	Description or example
Study details	Authors, date, title, journal, volume, issue, pages, country of origin,aim/objective of study
Description of the digital mental health resource	Purpose, technology type, target population, service type, therapeuticbasis, mode of delivery
***Variables relating to processes undertaken***	
Stage of development or evaluation	e.g. Predesign, early design, post first prototype, feasibility,efficacy or effectiveness trial, implementation
Methodology used	e.g. Participatory design, phenomenology, co-design, pilot study,randomised controlled trail
Participant demographics	e.g. Age, gender, ethnicity, languages spoken, diagnosis, role (i.e.student, patient, carer, health professional type)
Advisory boards	e.g. Leadership team, research group, consumer group
Data collection	Number and duration of design or evaluation sessions, sample size,sites of data collection (e.g. school, community service), methodsused (e.g. focus groups, workshops, interviews), support personnelincluded in design or evaluation processes (e.g. interpreters,support staff)
***Variables relating to research best practice***	
**Accessible, clear and co-constructed benefits to the community or individual**	
Justification for project and source	e.g. Literature, community consultation, previous formative studyor pilot
Dissemination practices	To whom, when, platforms used
**Meaningful relationships, consultation and participation of Indigenous people within all stages of research processes**	
Training or support provided to Indigenous research participants	e.g. Provision of tablet devices to test products, training in suicideprevention to aid design processes
Indigenous involvement in stages of research	e.g. Study design, funding, implementation, analysis, dissemination
Participant feedback on design or evaluation processes	e.g. Exit interview data, or rating scales of acceptability
Author reflections on design or evaluation processes	Reported results, strengths, limitations and recommendations
Research team experience in health research	Qualifications, time, reported relationships, credibility
**Respect and recognition of Indigenous knowledges and practices**	
Adaptions in processes in consideration of the physical,social, economic and cultural environment of participants	e.g. Community consent processes, interpreter involvement, followinglocal cultural protocols
Reporting and analysis which considers the physical, social,economic and cultural environment of the participants	e.g. Consideration of social determinants of health, strength-basedreporting
Rationale of methods/methodologies used	e.g. Literature, previous research, alignment with Indigenous worldviews
Consent processes reported	e.g. Individual, parent or collective consent, online or face to face,parties involved in consenting process (interpreter, support person)
**Indigenous self-determination and governance**	
Partnerships with Indigenous corporations or communities	e.g. Memorandum of Understandings, negotiation processes,agreements reached, approvals or agreements with location-specifichealth or governance boards
Ethics board clearances	e.g. Indigenous health research ethics committees

### Collating, summarising and reporting results

Relevant references will be exported into Endnote X9, including full text. Endnote X9 allows reviewers to collaboratively manage duplicates, group and code references and add annotations and notes. Data will be grouped by dMH intervention attributes, study design and alignment with research best practice and synthesised in table format. Qualitative content analysis will be undertaken [56]. Revision and refinement of themes will occur within the research team through a series of meetings. Results will be reported in narrative format, using tables to summarise important findings (e.g. dMH intervention attributes, study design and detail, alignment with best practice).

### Consultation

Emerging themes will be documented and used to facilitate discussion with two Senior Indigenous Research Officers (PPJRM) on at least three occasions, and written notes will be taken during consultation meetings [42]. Discussion between consultants and the research team will review and refine preliminary findings, aiming to reach consensus. This consultation phase, originally outlined by Arksey and O’Malley [44] and later refined and defined as necessary, by Levac et al. [42] enhances rigour, provides additional sources of information, perspectives, and meaning, and increases the applicability of research findings.

## Discussion

This scoping review aims to provide a comprehensive overview of current methods described in the literature regarding the involvement of Indigenous young people in development or evaluation of dMH interventions. The systematic nature of this review will ensure all relevant information on study methods is captured. Findings will provide insight into how methods align with best practice guidelines for the involvement of Indigenous people in ethical research and will inform recommendations for future projects aiming to develop or evaluate dMH interventions with Indigenous young people. As the design and use of dMH with Indigenous populations is an emerging field, potential limitations may be the small number of studies reported in the peer-reviewed literature as well as variations in study design and rigour. Therefore, broad definitions of inclusion terms will be used and the identification of relevant grey literature will expand data sources, allowing the identification of evidence gaps.

The findings from this review will assist researchers, clinicians and technology developers through establishing best practice processes for engagement of Indigenous young people in dMH approaches as well as for health research more broadly. As no other review has previously examined processes of development or evaluation in depth, this review aims to assist researchers to determine the ‘how’ of research design and develop clear methodology for respectful and culturally safe engagement with Indigenous young people.

## Supplementary Information

**Additional file 1.** PRISMA-P 2015 Checklist.

**Additional file 2.** Search terms.

## Data Availability

The materials supporting the article is included as Additional files 1 and 2.

## Abbreviations

CONSIDER: Consolidated criteria for strengthening reporting of health research involving Indigenous peoples
dMH: Digital Mental Health
PD: Participatory design
PRISMA-P: Preferred Reporting Items for Systematic Reviews and Meta-Analyses Protocols
PRISMA-Scr: Preferred Reporting Items for Systematic Reviews and Meta-Analyses extension for scoping reviews
