# Supplementary material for: Involvement of Indigenous young people in the design and evaluation of digital mental health interventions: a scoping review protocol
Source: Syst Rev. 2021 May 5;10:133. doi: 10.1186/s13643-021-01685-7 (PMC8101167; doi:10.1186/s13643-021-01685-7)
Supplement: Supplementary file 2 — Additional file 2. Search terms. [file 13643_2021_1685_MOESM2_ESM.docx]

# Search Terms EBSCOhost databases

**EBSCOhost databases: Selected databases:** Academic Search Premiere, Computer and Applied Science complete, CINAHL Plus with Full text, MEDLINE with full text, APA PsychArticles, Psychology and Behavioural sciences collection, APA PsychInfo.

| Search terms |
| --- |
| (“aboriginal and torres strait islander” or aboriginal or “first people” or “first nation people” or “first nation*” or indigenous or “Indian American” or metis or native or inuit or maori OR pacific* OR hawai* or alaskan)  **AND**  (youth or adolescen* or teenage* or “young people” or “young adult” or child*)  **AND**  (“e-Mental Health” OR e-Health OR “electronic health” OR “digital mental health” OR telemedic* OR telehealth OR “telemental health” OR mhealth OR “mobile health” OR mtherapy OR “online therapy” OR “online intervention” OR e-therapy OR “internet intervention” OR “computer-assisted therapy” OR “online self-help” OR iCBT OR “website intervention” OR “web-based treatment” OR “serious game*” OR SMS OR “text messaging” OR “text message” OR “distance based intervention” OR “internet-based intervention” or “teleintervention”)  **AND**  (“mental health” or psych* or wellbeing OR well-being OR distress) |

**Published date**: Jan 1990 – July 2020

**Resource types**: All results
